# Long-Term Depletion of Conventional Dendritic Cells Cannot Be Maintained in an Atherosclerotic Zbtb46-DTR Mouse Model

**DOI:** 10.1371/journal.pone.0169608

**Published:** 2017-01-06

**Authors:** Miche Rombouts, Nathalie Cools, Mandy O. J. Grootaert, Flore de Bakker, Ilse Van Brussel, An Wouters, Guido R. Y. De Meyer, Benedicte Y. De Winter, Dorien M. Schrijvers

**Affiliations:** 1 Laboratory of Physiopharmacology, Faculty of Pharmaceutical, Biomedical and Veterinary Sciences, University of Antwerp, Antwerp, Belgium; 2 Laboratory of Experimental Hematology, Vaccine and Infectious Disease Institute (Vaxinfectio), Faculty of Medicine and Health Sciences, University of Antwerp, Antwerp, Belgium; 3 Center for Oncological Research, Faculty of Medicine and Health Sciences, University of Antwerp, Antwerp, Belgium; 4 Laboratory of Experimental Medicine and Pediatrics, Faculty of Medicine and Health Sciences, University of Antwerp, Antwerp, Belgium; Universiteit van Amsterdam, NETHERLANDS

## Abstract

**Background and aims:**

Increased evidence suggests a pro-atherogenic role for conventional dendritic cells (cDC). However, due to the lack of an exclusive marker for cDC, their exact contribution to atherosclerosis remains elusive. Recently, a unique transcription factor was described for cDC, namely *Zbtb46*, enabling us to selectively target this cell type in mice.

**Methods:**

Low-density lipoprotein receptor-deficient (*Ldlr*^*-/-*^) mice were transplanted with bone marrow from *Zbtb46*-diphtheria toxin receptor (DTR) transgenic mice following total body irradiation. *Zbtb46*-DTR→*Ldlr*^*-/-*^ chimeras were fed a Western-type diet for 18 weeks while cDC were depleted by administering diphtheria toxin (DT).

**Results:**

Although we confirmed efficient direct induction of cDC death *in vitro* and *in vivo* upon DT treatment of *Zbtb46*-DTR mice, advanced atherosclerotic plaque size and composition was not altered. Surprisingly, however, analysis of *Zbtb46*-DTR→*Ldlr*^*-/-*^ chimeras showed that depletion of cDC was not sustained following 18 weeks of DT treatment. In contrast, high levels of anti-DT antibodies were detected.

**Conclusions:**

Because of the observed generation of anti-DT antibodies and consequently the partial depletion of cDC, no clear decision can be taken on the role of cDC in atherosclerosis. Our results underline the unsuitability of *Zbtb46*-DTR→*Ldlr*^-/-^ mice for studying the involvement of cDC in maintaining the disease process of atherosclerosis, as well as of other chronic inflammatory diseases.

## Introduction

The pathophysiological process of atherosclerosis is marked by chronic inflammation mediated by both innate and adaptive immune responses [1,2]. Dendritic cells (DC), the most potent antigen-presenting cells of the body, were found to be central in regulating these immune responses [3,4]. The presence of DC has been reported in atherosclerotic plaques [5–8], and dyslipidemia associated with atherosclerosis was found to alter DC activation and migration [9]. In addition, it was shown that vascular DC play a pivotal role in the earliest accumulation of lipids in the aortic wall [10], which forces us to reconsider the widely held view that monocytes are the earliest immune cells to contribute to lesion development. In the steady state, DC are essential in preserving arterial homeostasis by maintaining tolerance to self-antigens. However, after encountering modified antigens from the arterial wall under pro-inflammatory conditions, DC migrate into draining lymphoid tissues, where they may activate T cells specific for these antigens, thereby inducing and maintaining atherosclerotic inflammation [3,4]. The majority of murine DC belong to the conventional (c)DC subset, which can be further classified into cDC type 1, encompassing lymphoid tissue-resident CD8α^+^ cDC and their migratory counterparts CD103^+^ cDC, and CD11b^+^ cDC type 2 [11]. CD103^+^ cDC are likely tolerogenic and protect against atherosclerosis by regulating local homeostasis of regulatory T cells (Treg) [12]. Furthermore, we previously found that CD11b^+^ cDC are the most predominant subset in human plaques and that circulating numbers of CD11b^+^ cDC in mice strongly correlate with inflammation in early plaque development [13].

The past few years, researchers have attempted to clarify the specific contribution of cDC in atherosclerotic mice, with mixed or unsatisfactory results due to the lack of selective markers expressed on individual DC populations [10,14–16]. Recently, a novel and evolutionarily conserved zinc finger transcription factor was identified, *Zbtb46* (also known as *Btbd4* or zDC), which is exclusively expressed by pre-cDC, and lymphoid organ- and tissue-resident cDC, but not monocytes or other immune populations [17,18]. This has led to the development of a new mouse model in which the receptor for diphtheria toxin (DTR) was inserted into the 3’ untranslated region of the *Zbtb46* locus to serve as a way to specifically deplete cDC [17].

In the present study, we aimed at further elucidating the contribution of cDC, derived from pre-cDC, in the context of atherosclerosis. For this, lethally irradiated, atherosclerosis-prone, low-density lipoprotein receptor–deficient (*Ldlr*^*-/-*^) mice were transplanted with bone marrow from *Zbtb46*-DTR donor mice, allowing for specific depletion of cDC following administration of DT. The impact of this depletion was studied by analyzing immune cell distribution in blood and all relevant tissues, including spleen and mediastinal lymph nodes (LN).

## Materials and Methods

### Bone marrow transplantation and atherosclerosis induction in mice

Bone marrow chimeras were obtained through a bone marrow transplantation. Male and female 8–10 weeks old *Ldlr*^*-/-*^ recipient mice (Jackson Laboratory; stock number 002207) were exposed to a single dose of 10 Gray total body irradiation using an X-RAD 320 (Precision X-ray) 3h before reconstitution with 1×10^7^ bone marrow cells from *Zbtb46*-DTR transgenic mice (Jackson Laboratory; stock number 019506) via tail vein injection. Drinking water with antibiotics (0.15mg/ml Baytril^®^; Bayer) and 5g/l sucrose (Life Technologies) was introduced one week before irradiation until 3 weeks after transplantation. After a recovery period of 4 weeks, *Zbtb46*-DTR→*Ldlr*^*-/-*^ chimeras were fed a Western-type diet (WD) containing 0.2% cholesterol (4021.90; AB Diets) for 18 weeks.

### Treatment protocol

For transient cDC ablation, mice were injected intraperitoneally (i.p.) with 20ng diphtheria toxin (DT; Sigma-Aldrich) per gram of body weight (ng/g.bw). To maintain cDC ablation, *Zbtb46*-DTR→*Ldlr*^*-/-*^ chimeras received 4ng DT/g.bw on the 3rd day after the initial DT injection and every 3rd day thereafter. Control mice were injected with water for injection (NaCl 0.9%, Braun). At the end of the experiment, mice were euthanized with sodium pentobarbital (250mg/kg.bw, i.p.). All animals were housed in a temperature-controlled room with a 12h light/dark cycle and had free access to water and food. The animal procedures were performed conform the guidelines from Directive 2010/63/EU of the European Parliament on the protection of animals used for scientific purposes and all experiments were approved by the ethics committee of the University of Antwerp (permit number 2013–68).

### Determination of chimerism

Efficiency of the bone marrow transplantation was assessed as previously described by Kanters *et al*. [19]. In brief, qPCR analysis of the *Ldlr*^*-/-*^ gene was performed on bone marrow cells. Analysis of the household gene *P50* was used as a control. Purification of RNA was performed by means of an Absolutely RNA miniprep kit (Agilent technologies). For cDNA synthesis, a SuperScript II Reverse Transcriptase kit (Life Technologies) was used. A SensiMix SYBR Hi-ROX kit (Bioline) was used for SYBR Green-based qPCR. The *Ldlr*^*-/—*^and *P50*-specific primers are listed in Table 1. Samples were assayed in duplicate using a 7300 Real Time PCR System (Applied Biosystems). Data were analyzed using qBase+ software (Biogazelle).

**Table 1:** Primer sequences for determination of chimerism.

Gene	Sequence sense	Sequence antisense
***Ldlr***^**-/-**^	GCTGCAACTCATCCATATGCA	GGAGTTGTTGACCTCGACTCTAGAG
**P50**	AACCTGGGAATACTTCATGTGACTAA	GCACCAGAAGTCCAGGATTATAGC

Abbreviations: *Ldlr*^-/-^, low-density lipoprotein receptor-deficient; P50, DNA binding subunit of the NFκB protein complex.

### Culture of bone marrow-derived dendritic cells (BMDC)

Bone marrow cells from wild-type C57BL/6J mice (Jackson Laboratory; stock number 000664) and *Zbtb46*-DTR mice were isolated by flushing femurs and tibias. Cells were passed through a 40μm cell strainer and subjected to red blood cell lysis (Sigma-Aldrich). After two washing steps, 1×10^6^ per ml cells were seeded in 6-well plates in complete medium, consisting of RPMI 1640 medium supplemented with GlutaMAX (Life Technologies), 5% heat-inactivated FBS (Sigma-Aldrich), 1% penicillin/streptomycin (Life Technologies), 20U/ml polymyxin B (Fagron), 1mM sodium pyruvate (Life Technologies), 50μM β-mercaptoethanol (Life Technologies), and 10ng/ml GM-CSF (PeproTech). Cells were cultured at 37°C in a 5% CO_2_-humidified atmosphere for 6 days. At days 3 and 6, half of the culture medium was replenished. To study the effect of DT on immature BMDC, half of the cultured BMDC did not receive any maturation stimuli, and DT (2000ng/ml) was administered on day 6. On day 7, the remaining BMDC were activated by the addition of 1μg/ml lipopolysaccharide (LPS; Sigma-Aldrich) and 1000U/ml interferon-γ (IFN-γ; PeproTech), together with the addition of DT (2000ng/ml). Controls were not treated with DT. Twenty-four h after DT administration, cells were harvested for flow cytometric analysis.

### Flow cytometry

*Zbtb46*-DTR→*Ldlr*^*-/-*^ chimeras were euthanized and whole blood was obtained by cardiac puncture. The spleen and mediastinal LN were harvested, mechanically disaggregated and passed through a 40μm cell strainer to obtain single cell suspensions. Red blood cells were lysed, as described above, and leukocytes were counted using a hemocytometer. Subsequently, cells were resuspended in FACS buffer (PBS supplemented with 0.1% BSA (Sigma-Aldrich) and 0.05% NaN_3_ (Merck)), preincubated for 10min with Fc blocker (anti-mouse CD16/32 antibody; BioLegend), and then stained with fluorochrome-labeled antibodies for 30min at 4°C (Table 2). Cells were fixed and permeabilized prior to intracellular staining of heparin-binding epidermal growth factor-like growth factor (HB-EGF) receptor, also known as DTR. Cells were analyzed on a BD Accuri C6 cytometer (Becton Dickinson). Debris and dead cells were excluded based on light scatter properties and positive staining for propidium iodide (Invitrogen). A FITC Annexin-V apoptosis detection kit (BD Biosciences) was used for staining of apoptotic cells in cell cultures. Leukocyte subsets (Table 3) were analyzed using FCS Express 4 software (De Novo Software).

**Table 2:** Monoclonal antibodies used for flow cytometry.

Marker	Conjugate	Clone	Specificity	Source
**Anti-mouse antibodies**				
CD11c	APC	N418	cDC	BioLegend
MHCII	FITC	KH74	cDC, B cells	BioLegend
CD11b	PerCP	M1/70	cDC2, macrophages	BioLegend
CD103	PerCP-Cy5.5	2E7	cDC1	BioLegend
CD3ε	APC	145-2C11	T cells	BioLegend
CD19	PE	6D5	B cells	BioLegend
NK1.1	FITC	PK136	NK cells	BioLegend
Ly6C	APC	HK1.4	Monocytes	BioLegend
Gr-1	PE	RB6-8C5	Neutrophils	BioLegend
**Anti-human antibodies**				
HB-EGF	APC	IC259A	Diphtheria toxin receptor	R&D Systems

Abbreviations: cDC, conventional dendritic cells; HB-EGF, heparin-binding epidermal growth factor-like growth factor; MHCII, major histocompatibility complex class II alloantigen; NK cells, natural killer cells.

**Table 3:** Antibody combinations for defining leukocyte subsets by flow cytometry.

Cells	Antibody combination
**T cells**	CD3^+^NK1.1^-^
**NK cells**	CD3^-^NK1.1^+^
**B cells**	CD19^+^
**neutrophils**	CD11b^+^Gr-1^high^
**monocytes**	Ly-6C^low^ or Ly-6C^high^
**cDC**	CD11c^+^MHCII^+^, either CD11b^+^ or CD103^+^

### Total plasma cholesterol

Analysis of total plasma cholesterol was performed by using a colorimetric assay (Randox) according to the manufacturer’s instructions.

### Histology and immunohistochemistry

At sacrifice, the aortic root was collected, embedded in Neg-50 (Thermo Scientific) and snap frozen in liquid nitrogen. Atherosclerotic plaque size was analyzed on Oil-Red-O (Sigma-Aldrich) stained cryosections and the necrotic core was analyzed on haematoxylin-eosin (H-E) stained sections as the acellular area with a threshold of 3000 μm^2^.[20] Collagen content was measured by Sirius red (Sigma-Aldrich) staining. Immunohistochemical staining was performed for α-smooth muscle actin (α-SMA; Sigma-Aldrich), CD11c (Abcam) and for cleaved caspase-3 (Cell Signaling Technologies).

A shortened version of the Llewellyn protocol was applied to the spleen before staining with antibodies [21]. Cryosections were double stained with rat anti-CD68 (AbD Serotec), rat anti-Ter-119 (BD Pharmingen), rat anti-CD31 (BD Pharmingen), or Armenian hamster anti-CD11c combined with rabbit anti-cleaved caspase-3. Sections were mounted with Vectashield mounting medium containing DAPI (Vector Laboratories) and visualized by fluorescence microscopy. All other images were acquired with Universal Grab 6.1 software using an Olympus BX40 microscope and quantified with ImageJ software (National Institutes of Health).

### ELISA

An in-house ELISA, based on Buch et al.[22], was developed to measure the presence of anti-DT-specific IgG antibodies in plasma samples. In brief, Nunc Maxisorb 96-well plates were coated with 2μg/ml DT antigen (Sigma-Aldrich) and subsequently blocked with 5% non-fat dry milk (Bio-Rad). Plasma samples were applied to the 96-well plates in 5-fold dilution and the presence of bound antigen-specific IgG_1_ antibodies was detected using a 1:50,000 dilution of a peroxidase-conjugated secondary rabbit anti-mouse IgG antibody (Sigma-Aldrich). A monoclonal antibody recognizing DT (7F2, IgG_1_ isotype; Abcam) was used as a positive control. Colorimetric development following addition of peroxidase substrate (TMB; eBioscience) was quantified as optical density at 450nm (OD_450_) using a spectrophotometer (Bio-Tek).

### Statistical analysis

Statistical analysis was performed using SPSS Statistics 23.0 (IBM) by means of Student t test or One-way ANOVA followed by Tukey’s Multiple Comparison post-hoc test, as appropriate. Univariate analyses were performed for plaque area and composition of aortic root sections. Data that failed the Levene's test of homogeneity of variances were mathematically transformed before statistical analysis was performed. Graphs were created with GraphPad Prism 6 (GraphPad Software). Data are shown as mean ± SEM. Differences were considered significant when *p*<0.05.

## Results

### The Zbtb46-DTR mouse model enables cDC depletion both *in vitro* and *in vivo*

**Fig 1:**
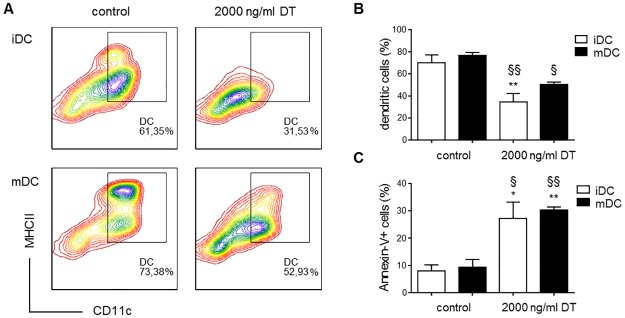
*In vitro* validation of the *Zbtb46*-DTR model to deplete cDC. (A,B) Representative CD11c/MHC class II contour plots (A) and quantification of flow cytometric analysis (B) of *in vitro* immature (iDC) and mature (mDC) BMDC from *Zbtb46*-DTR mice treated with 2000ng/ml DT for 24h or left untreated (control) (n = 3–4); (C) Quantification of *in vitro* cultures of BMDC from *Zbtb46*-DTR mice labeled with FITC Annexin-V for the detection of apoptotic cells after no treatment (control) or treatment with DT (2000ng/ml, 24h) (n = 3–4); Statistical analysis was performed by means of One-way ANOVA followed by a Tukey’s Multiple Comparison post-hoc test; *p<0.05, **p<0.01, significantly different from control iDC; ^§^*p*<0.05, ^§§^*p*<0.01, significantly different from control mDC.

To validate the sensitivity of cDC to DT and the competence of the *Zbtb46*-DTR mouse model to deplete cDC, *in vitro* and *in vivo* experiments were performed. Treatment of BMDC of *Zbtb46*-DTR mice with 2000ng/ml DT resulted in a significantly lower proportion of immature and mature cDC (Fig 1A and 1B) that could be recovered from the *in vitro* cell culture. This reduction was accompanied by a concomitant increase in Annexin-V-positive cells (Fig 1C). By contrast, immature and mature BMDC from wild-type mice were insensitive to DT treatment, as evidenced by similar proportions of Annexin-V-positive cells following DT treatment (S1 Fig).

**Fig 2:**
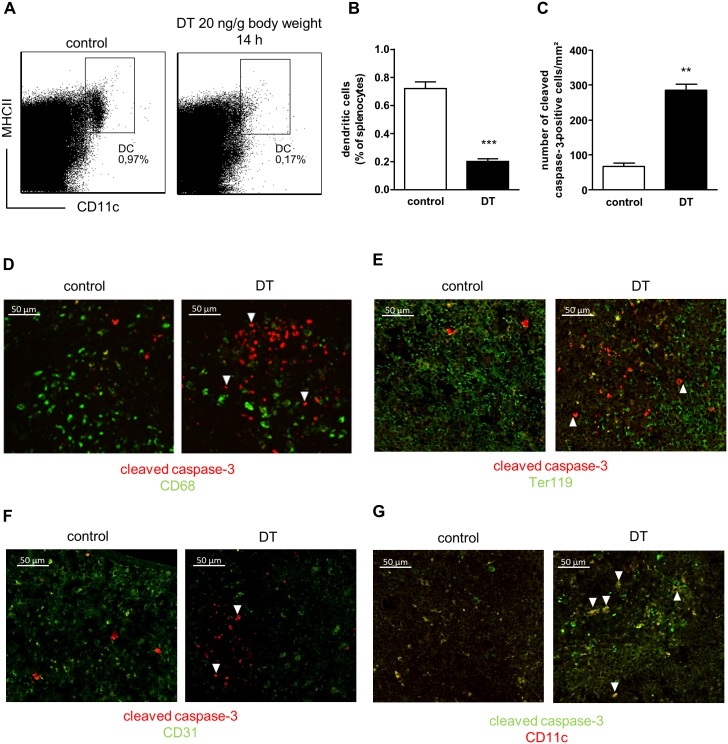
*In vivo* validation of the *Zbtb46*-DTR model to deplete cDC. (A,B) Representative dot plots (A) and flow cytometric analysis (B) of splenocytes of *Zbtb46*-DTR mice sacrificed 14h after a single injection with vehicle (control, n = 6) or 20ng DT/g.bw (n = 5); Differences between groups were analyzed by means of a Student t test, ***p<0.001; (C) Quantification of cleaved caspase-3 positivity in spleens (average of 3 measurements per mouse, n = 2 mice per group); Differences between groups were analyzed by means of a Student t test, **p<0.01; (D-F) Cleaved caspase-3 (red, arrow heads) and (D) CD68, (E) CD31, and (F) Ter119 staining of spleens from *Zbtb46*-DTR mice treated with vehicle (control) or DT (20ng/g.bw, 14h); (G) Double staining of spleens from control or DT-treated *Zbtb46*-DTR mice with cleaved caspase-3 (green) and CD11c (red). Arrow heads indicate apoptotic CD11c^+^ cells; Scale bar = 50μm.

The *in vivo* experiment was performed following the method described by Meredith *et al*. [17]. In brief, *Zbtb46*-DTR mice were sacrificed 14h after a single injection with vehicle or 20ng DT/g.bw. Flow cytometric analysis of splenocytes demonstrated a significant drop (72%) in the percentage of total cDC 14h after DT injection (Fig 2A and 2B). As part of the *in vivo* validation of the *Zbtb46*-DTR model, a cleaved caspase-3 staining was applied to a limited number of spleens. Quantification of the number of cleaved caspase-3-positive cells showed a significant difference between the two groups, thereby confirming elevated cell death *in vivo* after DT administration (Fig 2C). Interestingly, the cells undergoing apoptosis are not macrophages, committed erythroid progenitors or endothelial cell populations [18], as cells positive for CD68, Ter119, and CD31 do not co-express caspase-3 (Fig 2D–2F). Double staining of cleaved caspase-3 and CD11c, which is used here as a pan marker for DC, revealed the presence of apoptotic CD11c^+^ cells in spleens of DT-treated mice (Fig 2G, arrow heads). However, it needs to be noted that apoptosis of CD11c^+^ cells could also be detected in the control samples, albeit CD11c staining in these samples was not optimal.

### Long-term DT treatment does not affect the leukocyte profile in blood, spleen and LN

Similar to CD11c-DTR mice, it is described that a single injection of DT is lethal in *Zbtb46*-DTR mice within 24-48h, probably due to the expression of *Zbtb46* in committed erythroid progenitors and endothelial cells. Therefore, experiments involving prolonged DT administration necessitate the use of radiation chimeras in which non-hematopoietic cells remain of recipient origin, insensitive to DT [17,23]. In this study, lethally irradiated *Ldlr*^*-/-*^ mice were grafted with bone marrow cells from *Zbtb46*-DTR mice to generate *Zbtb46*-DTR→*Ldlr*^*-/-*^ chimeras. A degree of chimerism of >90% was considered successful. On average, a chimerism degree of 94.1 ± 2.4% was achieved.

**Fig 3:**
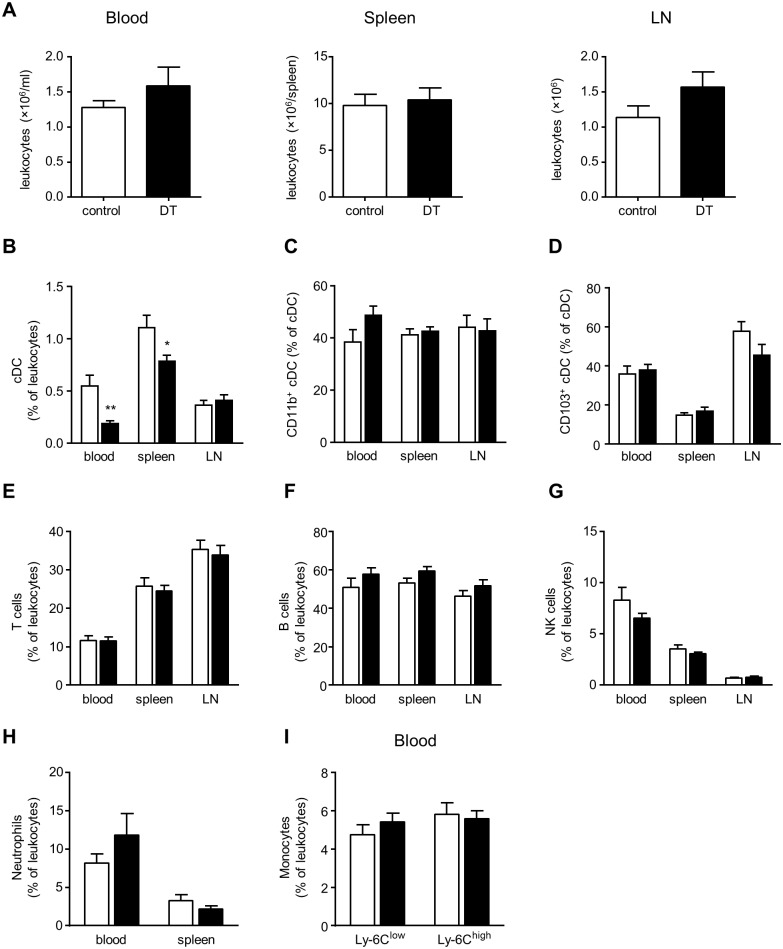
Long-term DT treatment of *Zbtb46*-DTR→*Ldlr*^*-/-*^ mice does not alter cDC or leukocyte numbers. (A) Absolute numbers of leukocytes after 18 weeks of WD feeding in vehicle (control) and DT-treated *Zbtb46*-DTR→*Ldlr*^*-/-*^ mice in blood, spleen, and LN (n = 17–19); (B-D) Flow cytometric analysis of total cDC (B) and CD11b^+^ (C) and CD103^+^ (D) cDC subsets in blood, spleen, and LN of control and DT-treated *Zbtb46*-DTR→*Ldlr*^*-/-*^ mice (n = 14–19); (E-I) Flow cytometric analysis of T cells (E), B cells (F), NK cells (G), and neutrophils (H) in blood, spleen and LN, and of Ly-6C^low/high^ monocyte subsets (I) in blood of control and DT-treated *Zbtb46*-DTR→*Ldlr*^*-/-*^ mice (n = 16–19); Statistical analysis was done by means of a Student t test: *p<0.05, **p<0.01.

For long-term administration of DT (18 weeks), the treatment protocol was followed as described by Meredith *et al*. [17]. In brief, *Zbtb46*-DTR→*Ldlr*^*-/-*^ chimeric mice were injected every 3 days with 4ng/g.bw DT. Upon sacrifice, blood, spleen, and mediastinal LN were collected for flow cytometric analysis (S2 Fig, gating strategy). Total leukocyte numbers in blood, spleen, and LN did not differ between the control and treatment group (Fig 3A). Total cDC were significantly reduced from blood and spleens of DT-treated *Zbtb46*-DTR→*Ldlr*^*-/-*^ chimeric mice (Fig 3B). No differences could be seen in cDC numbers in the LN, nor in CD11b^+^ and CD103^+^ cDC subsets (Fig 3C and 3D). As leukocytes crucially affect atherosclerosis, we examined the effect of chronic DT treatment of *Zbtb46*-DTR→*Ldlr*^*-/-*^ chimeras on other leukocyte subpopulations as well. Flow cytometric analysis of blood, spleen and LN did not reveal any significant differences in the proportion of T cells, B cells, NK cells or neutrophils (Fig 3E–3H). In addition, the percentage of Ly-6C^low^ and Ly-6C^high^ monocytes in blood did not differ between the control and treatment group (Fig 3I).

### Chronic DT administration does not affect atherosclerosis in Zbtb46-DTR→Ldlr^-/-^ mice

In parallel with the flow cytometric analysis, we examined whether long-term DT administration had an impact on atherosclerosis. Therefore, *Zbtb46*-DTR→*Ldlr*^*-/-*^ chimeric mice were fed a WD diet while injected with 4ng/g.bw DT every 3 days for 18 weeks. DT administration was well tolerated, with no differences in body weight between the control and experimental group (Table 4). Noteworthy, as a result of the irradiation therapy, the fur of all mice grayed. Analysis of the spleen weight revealed a significant decrease in DT treated mice. There was no significant difference for heart weight between the two groups (Table 4). Furthermore, plasma cholesterol levels did not differ between the control and DT-treated group (Table 4).

**Table 4:** Weight and plasma characteristics from control and DT-treated *Zbtb46*-DTR→*Ldlr*^-/-^ mice.

	Control	DT
Body weight (g)	26±1	27±1
Spleen weight (mg)	133±9	108±7*
Heart weight (mg)	130±4	116±3
Plasma cholesterol (mg/dl)	734±57	755±38

Data from vehicle (control) and DT-treated *Zbtb46-DTR*→*Ldlr*^-/-^ mice after 18 weeks of WD feeding, mean ± SEM, control n = 19, DT n = 16–17; Differences between groups were analyzed by means of a Student t test,

*p<0.05.

**Fig 4:**
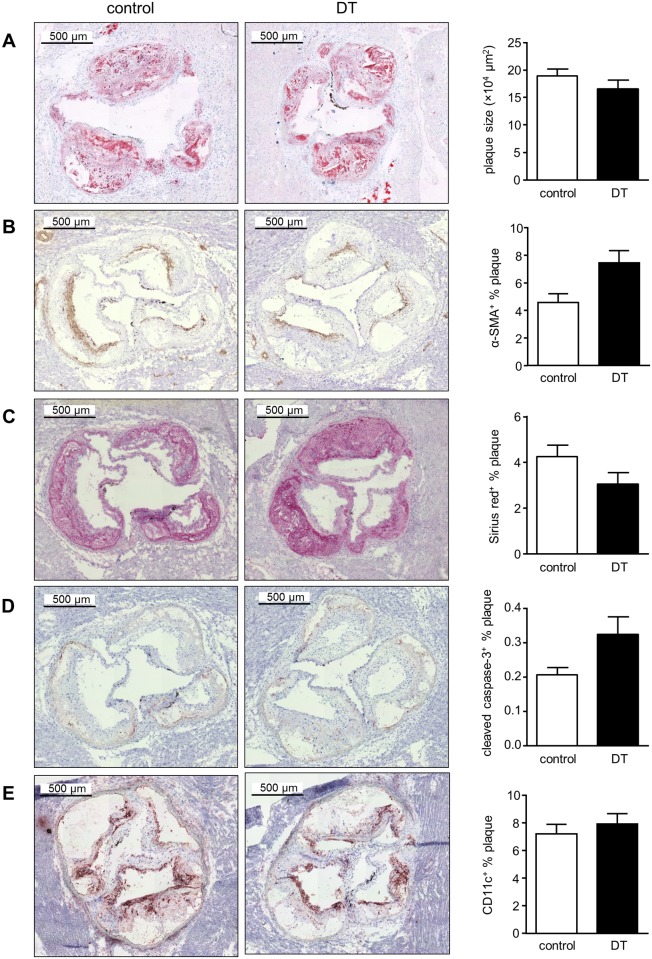
Chronic DT administration does not affect atherosclerotic plaque size and composition in *Zbtb46*-DTR→*Ldlr*^*-/-*^ mice. Representative images and quantification of (A) atherosclerotic lesion size (Oil Red O^+^ area), (B) vascular smooth muscle cells (α-SMA staining), (C) collagen (Sirius Red staining), (D) apoptosis (cleaved caspase-3 staining), and (E) CD11c^+^ cells in aortic root cryosections of control and DT-treated *Zbtb46*-DTR→*Ldlr*^*-/-*^ mice (n = 14–19); Univariate analyses were performed for plaque area and composition of aortic root sections. Data that failed the Levene's test of homogeneity of variances were mathematically transformed before statistical analysis was performed.

Oil-Red-O stained cryosections were used to determine the plaque area. Plaques in the aortic root did not show a difference in size between control and DT-treated chimeras (Fig 4A). Features of plaque stability were similar in both groups, as no changes were observed in vascular smooth muscle cell content and total collagen (Fig 4B and 4C). Moreover, the degree of necrosis did not differ between control (25.28 ± 2.56%) and DT-treated mice (27.48 ± 2.71%) (*p* = 0.792). DT binds to HB-EGF (which acts as DTR) and, following internalisation, inhibits protein synthesis which rapidly induces apoptosis [24]. Nonetheless, staining of plaques with cleaved caspase-3 antibody did not show a significant increase in plaque apoptosis between control and DT-treated chimeras (Fig 4D). Consequently, no difference was observed in the proportion of CD11c^+^ cells within plaques (Fig 4E).

### Insufficient cell depletion is mediated by anti-DT humoral immunity, but independent of DTR resistance

**Fig 5:**
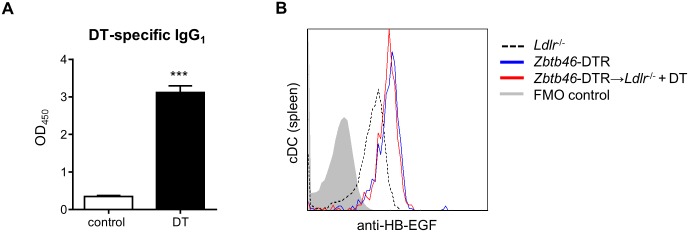
Insufficient cDC depletion is mediated by DT-specific humoral immunity, but independent of DTR resistance. (A) DT-specific IgG_1_ antibodies were determined in plasma samples from control (n = 16) and DT-treated mice (n = 11) by ELISA and are shown as OD_450_ values; Statistical significance was determined by means of a Student t test, ***p<0.001; (B) Representative histogram of intracellular human DTR expression (anti-HB-EGF) assessed by flow cytometry of splenic cDC in *Ldlr*^-/-^ mice (dotted black), *Zbtb46*-DTR mice (blue) and *Zbtb46*-DTR→*Ldlr*^*-/-*^ mice (DT-treated, red) at the end of the study. An FMO sample is used as control (grey).

Since the efficacy of DT-mediated cell ablation may be hampered by the development of neutralizing anti-DT antibodies upon repeated administration of DT [22], we determined the presence of antibodies directed against DT in plasma samples of untreated (control) and DT-treated mice. Indeed, DT-specific antibodies of the IgG_1_ isotype were detected after 18 weeks of treatment with DT (OD_450_ 3.12±0.18 *vs*. 0.35±0.02 in control mice; Fig 5A), which could account for the ineffectiveness of this mouse model to achieve long-term cDC depletion using the DT-mediated technology.

Previously, it was also described that following continuous DT treatment a cell population lacking DTR expression and hence resistant to further DT-mediated depletion emerged [25,26]. In order to exclude the possibility that the lack of an effect on atherosclerosis development is due to the loss of DTR expression on DC, we compared intracellular DTR expression by flow cytometric analysis between splenic cDC from *Zbtb46*-DTR mice before DT treatment and splenic cDC from *Zbtb46*-DTR→*Ldlr*^*-/-*^ chimeras at the end of the study. Splenic cDC from *Ldlr*^-/-^ mice were used as a control. As shown in Fig 5B, splenic cDC from *Zbtb46*-DTR mice before DT treatment as wells as from *Zbtb46*-DTR→*Ldlr*^*-/-*^ chimeras after chronic DT treatment robustly express intracellular DTR.

## Discussion

Up till now, investigating the contribution of cDC to atherosclerosis was hampered by the fact that there is no selective marker exclusively expressed by cDC. Nevertheless, depletion of DC was first achieved by Jung *et al*. who used the murine CD11c promoter for the transgenic expression of human DTR [14]. When bred onto the *Ldlr*^*-/-*^ background, DT treatment resulted in a marked reduction in intimal CD11c^+^ cells and a 55% decrease in accumulation of lipids during the earliest stages of plaque formation [10]. Prolonging the lifespan of DC (and CD11c-expressing macrophages) in mice that carry CD11c-specific expression of the anti-apoptotic hBcl2, did not accelerate plaque progression [16]. Moreover, cholesterol levels were reduced in these mice, whereas depletion of DC resulted in enhanced cholesterolemia, indicative of a potential role for DC in cholesterol homeostasis [16]. Nevertheless, a major drawback of using the CD11c-DTR mouse model is the depletion of other immune cells besides DC, also expressing CD11c such as metallophilic and marginal zone macrophages [27]. Depletion of monocytes and macrophages in CD11b-DTR transgenic mice was shown to differentially affect atherosclerosis. Unfortunately, the authors did not investigate whether the CD11b^+^ cDC subpopulation was also depleted [28]. Constitutive deficiency of the chemokine receptor CX_3_CR1 in apolipoprotein E–deficient (*ApoE*^*-/-*^) mice resulted in an impaired accumulation of DC in the aortic wall and reduced atherosclerotic burden at early stages [29]. However, CX_3_CR1 is also required for monocyte recruitment and survival.

Here, we used the recently developed *Zbtb46*-DTR mouse model which allows specific depletion of cDC and their precursors, while sparing other hematopoietic cells [17,18]. *Zbtb46* acts as a suppressor of DC activation and MHC class II expression in the immature state [30]. In the present study, we aimed at dissecting the exact role of cDC in the context of atherosclerosis. In analogy to the experiments conducted by Meredith *et al*. [17], our *in vitro* and *in vivo* validation experiments confirmed that cDC could be depleted upon injection of DT in a *Zbtb46*-DTR mouse model. However, we were not able to reach the same degree of depletion as reported by Meredith *et al*. [17], who demonstrated a near complete ablation of cDC in spleens of DT-treated *Zbtb46*-DTR mice. Here, depletion of cDC could not be fully sustained for a longer period of time in *Zbtb46*-DTR→*Ldlr*^*-/-*^ chimeras, despite continuous DT injections. Except for a partial depletion in blood and spleen, no depletion of cDC was observed in LN after 18 weeks of DT treatment. In most studies reported so far, only short term DT administration is used to evaluate depletion efficiency *in vitro* and *in vivo*. In general, studies examining atherosclerosis in DTR mouse models for more than 2 weeks are scarce and often do not detail on DC numbers in the circulation or associated lymphoid tissues. On the basis of previously published reports regarding the kinetics of DC depletion in DTR transgenic mice, a twice-weekly DT administration regimen was chosen for the long-term depletion of cDC in *Zbtb46*-DTR→*Ldlr*^*-/-*^ chimeras [14,17]. However, following 18 weeks of DT treatment, anti-DT antibodies arise in the circulation of treated animals, possibly explaining the inefficient depletion in this study, in agreement with a recent study by Wang *et al*.[31]. Although even incomplete depletion in CD11c-DTR mice (with 70–80% efficacy) was reported to alter the balance of the immune response [32], and decrease inflammatory cytokine production in atherosclerosis [33] by others, we could not confirm these findings. For this, we hypothesize that the remaining DC potently stimulate the immune response, and that consequently any partial depletion would not be sufficient to suppress the long-term development of atherosclerosis in *Zbtb46*-DTR→*Ldlr*^-/-^ chimeras. While anti-DT antibody levels should be carefully monitored when DT treatment is used for more than 2 weeks, at the same time efforts should be made to ascertain whether the development of anti-DT antibodies can be avoided, be reduced or delayed, such as less frequent administration or lower dose of DT.

Very recently, an inducible system for cDC depletion was developed by Loschko *et al*. [34] who introduced a loxP-flanked transcriptional Stop element in front of the DTR, and inserted this cassette into the *Zbtb46* gene (zDC^lSlDTR^). To restrict DTR expression to cDC, zDC^lSlDTR^ mice were crossbred with transgenic mice expressing Cre under the control of the *Csf1* receptor gene (*Csf1r*^Cre^) [34]. In *Csf1r*^Cre+^zDC^lSlDTR^ mice expression of DTR under the control of *Zbtb46* is restricted to cDC which bypasses endothelial cells and omits the use of bone marrow chimeras. cDC can be efficiently depleted for at least 4 weeks in these mice. However, as previously demonstrated [35], the authors reported increased levels of Flt3L upon cDC depletion, whereas it is not known if anti-DT immunity occurs in these mice upon repeated administration of DT. Altogether, it is unclear from these observations if cDC depletion is sustained longer than 4 weeks.

Although in our hands incomplete depletion does not impact atherosclerosis, this is not always the case. Others reported that DT treatment of *ApoE*^-/-^CD11c-DTR bone marrow chimeras resulted in a significant DC depletion of approximately 78% in the aorta and spleen. As a result, transient depletion reduced oxLDL uptake and cytokine expression in the aortas of DT-injected mice. However, these mice were fed a WD for 12 weeks, of which they were injected with DT during the last 2 weeks, whereas in the present study, we started DT treatment before the onset of atherosclerosis and repetitively injected DT up to 18 weeks [33]. Albeit that it is possible that an 18-week follow up period is too long in order to observe an effect on plaque development, because these mice are already in a more progressive stage of disease, it would be interesting to longitudinally investigate the role of cDC in the initiation of atherosclerotic plaque development at earlier time points of the disease before the onset of anti-DT antibodies.

Others recently suggested the emergence of targeted cell populations that have lost DTR expression as an explanation for insufficient cell depletion in DTR models [25,26]. However, our results demonstrate that, even after long-term DT treatment, the receptor for DT is present on splenic cDC to the same extent as on splenic cDC from untreated *Zbtb46*-DTR mice. Zbtb46 expression was shown to be restricted to cDC precursors (pre-cDC) and lymphoid organ- and tissue-resident cDC. However, Satpathy *et al*. [18] demonstrated that a major subpopulation of pre-cDC in the bone marrow expressed the plasmacytoid (p)DC marker SiglecH, and that by inserting a GFP reporter cassette into the Zbtb46 locus, four distinct pre-cDC populations could be defined by Zbtb46 *vs*. SiglecH expression. Importantly, they showed that, although SiglecH is specific for pDC in peripheral organs, bone marrow progenitors expressing SiglecH but not Zbtb46, retain the potential to develop into cDC [18]. Hence, although in the *Zbtb46*-DTR mouse model cDC and Zbtb46-expressing pre-cDC are depleted, other pre-cDC capable to replenish cDC in the periphery may exist in the bone marrow. In this perspective others demonstrated that *Zbtb46* deficiency alters the cDC subset composition in the spleen in favor of CD8^+^ cDC [30]. Whether this could have contributed to the immune-mediated pathogenesis seen here should be further examined. Nevertheless, we found no differences in the proportions of CD11b^+^ and CD103^+^ cDC between control and DT-treated mice. Additionally, *Zbtb46*-DTR mice provide a model to ablate pre-DC–derived cDC while sparing other hematopoietic cells [17]. However, as a consequence of hypercholesterolemia, blood monocytes can be recruited into the intima where they can give rise to phenotypically similar monocyte-derived CD11b^+^ DC populations making it difficult to observe a relative contribution of cDC derived from pre-DC versus those derived from monocytes in the context of atherosclerosis. Yet, we did not observe any changes in circulating monocyte subsets.

Taken together, as a possible consequence of the development of anti-DT antibodies, *Zbtb46*-DTR→*Ldlr*^*-/-*^ mice become resistant to DT treatment during 18 weeks resulting in a partial recovery of the cDC. For this, we cannot pinpoint the role of cDC in atherosclerosis and conclude that the *Zbtb46*-DTR→*Ldlr*^-/-^ mouse model is not suitable to study the involvement of cDC in atherosclerosis, or in other chronic inflammatory diseases. Whether or not *bona fide* cDC significantly contribute to atherosclerotic plaque formation and stability remains to be determined.

## Supporting Information

S1 Fig Immature and mature BMDC from C57BL/6J wild-type mice are insensitive to DT treatment. (A) Representative CD11c/MHCII contour plots of in vitro immature (iDC) and mature (mDC) BMDC from C57BL/6J wild-type mice treated with 2000ng/ml DT for 24h or left untreated (control); (B) Quantification of flow cytometric analysis of BMDC from C57BL/6J mice treated with DT (2000ng/ml, 24h) or left untreated (control) (n = 2). (C) Quantification of in vitro cultures of BMDC from C57BL/6J wild-type mice labeled with FITC Annexin-V for the detection of apoptotic cells after no treatment (control) or treatment with DT (2000ng/ml) for 24h (n = 2).
(TIF)

S2 Fig Gating strategy for the analysis of immune cells in blood, spleen and LN of *Zbtb46*-DTR→*Ldlr*^*-/-*^ chimeras. Gates are set on isotypes to correct for non-specific binding. (A) Plots are pre-gated on FSC and SSC to define total leukocytes from cell debris. (B) The total cDC population was identified based on the expression of CD11c^(high)^ and MHC class II. (C,F) Based on their expression of CD11b (C) and CD103 (F) two cDC subsets were identified. (D) A distinction was made between circulating Ly-6C^low^ and Ly-6C^high^ monocytes in blood. Lymphocyte subsets were identified as (E) T cells (CD3^+^ NK1.1^-^), NK cells (CD3^-^ NK1.1^+^) and (G) B cells (CD19^+^). (H) Neutrophils were identified as CD11b^+^ Gr-1^high^ cells.
(TIF)
