# Supplementary figures and images for: Long-Term Depletion of Conventional Dendritic Cells Cannot Be Maintained in an Atherosclerotic Zbtb46-DTR Mouse Model
Source: PLoS One. 2017 Jan 6;12(1):e0169608. doi: 10.1371/journal.pone.0169608 (PMC5218565; doi:10.1371/journal.pone.0169608)

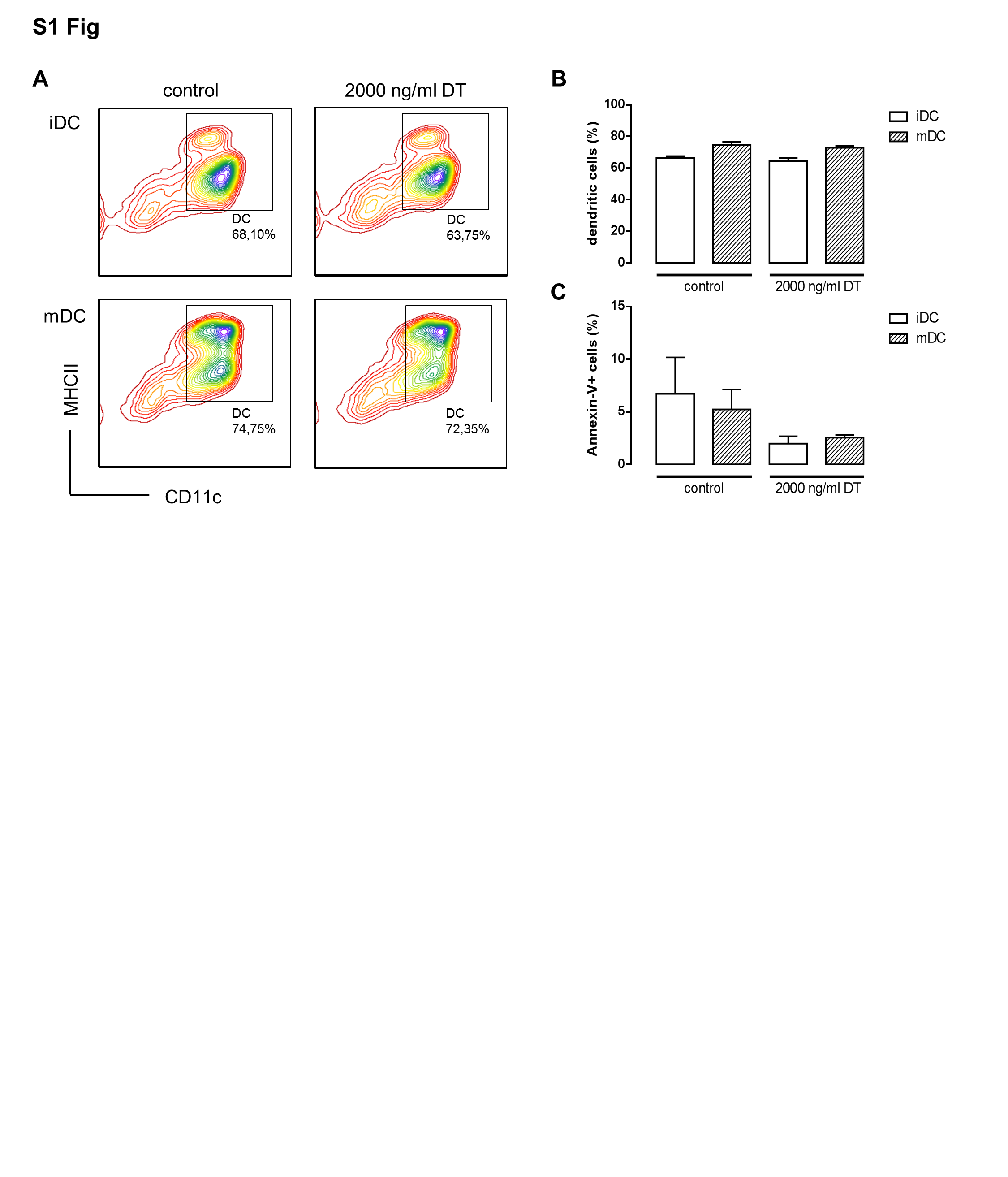

Supplement: S1 Fig — (A) Representative CD11c/MHCII contour plots of in vitro immature (iDC) and mature (mDC) BMDC from C57BL/6J wild-type mice treated with 2000ng/ml DT for 24h or left untreated (control); (B) Quantification of flow cytometric analysis of BMDC from C57BL/6J mice treated with DT (2000ng/ml, 24h) or left untreated (control) (n = 2). (C) Quantification of in vitro cultures of BMDC from C57BL/6J wild-type mice labeled with FITC Annexin-V for the detection of apoptotic cells after no treatment (control) or treatment with DT (2000ng/ml) for 24h (n = 2). (TIF) [file pone.0169608.s001.tif]

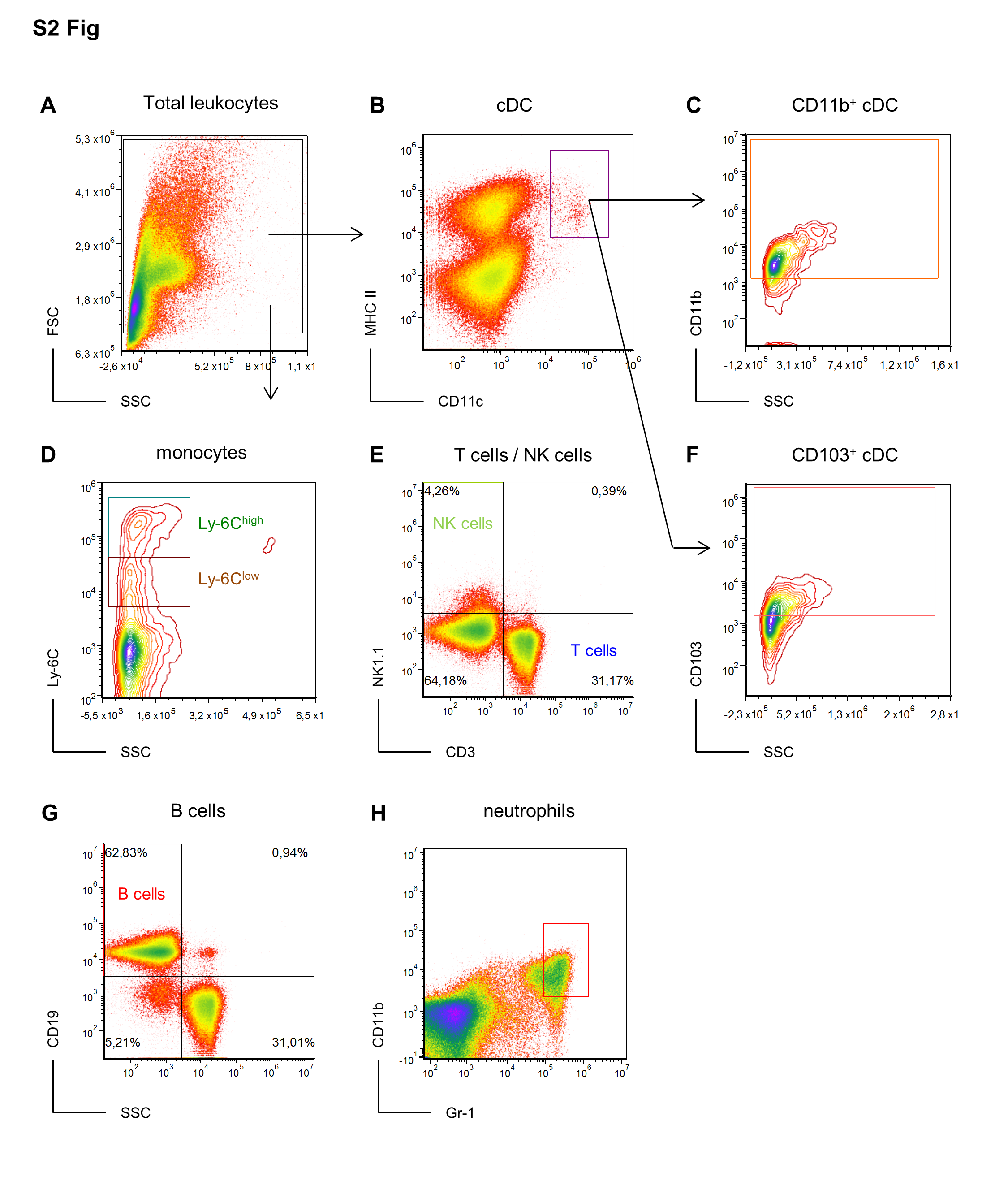

Supplement: S2 Fig — Gates are set on isotypes to correct for non-specific binding. (A) Plots are pre-gated on FSC and SSC to define total leukocytes from cell debris. (B) The total cDC population was identified based on the expression of CD11c(high) and MHC class II. (C,F) Based on their expression of CD11b (C) and CD103 (F) two cDC subsets were identified. (D) A distinction was made between circulating Ly-6Clow and Ly-6Chigh monocytes in blood. Lymphocyte subsets were identified as (E) T cells (CD3+ NK1.1-), NK cells (CD3- NK1.1+) and (G) B cells (CD19+). (H) Neutrophils were identified as CD11b+ Gr-1high cells. (TIF) [file pone.0169608.s002.tif]
